# Adaptive Laboratory Evolution Unlocks Membrane Permeability as a Key Limitation in Long‐Chain Alcohol Metabolism by *Pseudomonas putida* KT2440

**DOI:** 10.1111/1751-7915.70399

**Published:** 2026-06-23

**Authors:** Raul Mireles, Lianet Noda‐García

**Affiliations:** ^1^ Department of Plant Pathology and Microbiology, Institute of Environmental Sciences, The Robert H. Smith Faculty of Agriculture, Food and Environment The Hebrew University of Jerusalem Rehovot Israel

## Abstract

*Pseudomonas putida* KT2440, renowned for its diverse metabolic capabilities, is a promising platform for downstream processing and revalorisation of recalcitrant molecules. In this study, we examined and optimised *P. putida* KT2440's ability to utilise products of the degradation of polyethylene (PE), the most used and disposed plastic. PE degradation creates over 200 molecules that vary in oxidation level and, thus, chemical properties. Among those, long‐chain alcohols represent one of the most challenging fractions to process due to their poor solubility. Using them as feedstock for microbial growth would close the plastic‐derived carbon cycle, reducing environmental impact. First, we discovered that *P. putida* KT2440 can use the long‐chain alcohols, 1‐hexadecanol and 1‐eicosanol, as the sole carbon and energy source. Using adaptive laboratory evolution (ALE), we generated variants with improved growth rates on such substrates. Mutations that became fixed during ALE provided insights into the mechanism, highlighting the importance of cell–substrate interaction. By heterologously expressing a hydrocarbon transporter‐encoding gene, we successfully reproduced the ALE‐derived phenotype, suggesting that the bottleneck in long‐chain alcohol utilisation lies in uptake rather than substrate transformation. These findings lay the groundwork for the potential application of *P. putida* KT2440 for the valorisation of PE degradation products.

## Graphical abstract

*Pseudomonas putida* KT2440 grows on polyethylene‐derived long‐chain alcohols (C16OH, C20OH) as sole carbon sources. Adaptive laboratory evolution rapidly increases growth, converging on envelope‐associated mutations. Expressing the outer‐membrane transporter AlkL recapitulates much of the evolved phenotype, identifying substrate access and membrane permeability as key engineering levers for plastic‐waste valorisation in practice.

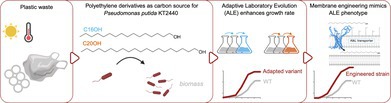

## 1 Introduction

Plastics are an integral part of contemporary life (Geyer et al. 2017). Their low production costs and versatile functionality made them ubiquitous across all economic sectors (e.g., from the food industry to healthcare) (OECD 2022a). This demand has driven global plastics production to an average of ~400 million metric tons (Mt) per year over the last 5 years, a market that is projected to produce 1231 Mt yearly by 2060 (PlasticsEurope 2025; OECD 2022a). This increasing demand is accompanied by inadequate disposal. Post‐use of this waste is mostly landfilled (46%), dumped (22%) or incinerated (17%), with only 15% collected for recycling (OECD 2022a). These shares highlight a persistent gap between consumption and end‐of‐life capacity to fulfil the circularity of plastics.

Among plastics, polyethylene (PE) is the main contributor to production (110 Mt, 24%) and waste (93.9 Mt, 26.6%) (OECD 2022b). PE is an oil‐derived polymer composed of strong interacting linear chains with C‐C and C‐H bonds, making it highly resistant to degradation (Schwab et al. 2024). Upon environmental discharge, PE films have an estimated half‐life of 2500 years (Chamas et al. 2020). While the impact of discarded PE in the environment is yet to be fully understood, early evidence points to greenhouse‐gas emissions from weathering, release of dissolved organic carbon that shifts carbon cycling, vectoring of pollutants and physical smothering that restricts light and oxygen, degrading habitat quality (Royer et al. 2018; Romera‐Castillo et al. 2018; Koelmans et al. 2016; Green et al. 2015). Proper disposal of PE is of paramount importance.

PE recycling efforts have focused on developing strategies to break down the polymer into simpler molecules. These transformations, whether thermo‐, photo‐ or chemically catalysed, are largely non‐selective and typically yield complex product mixtures. So far, over 200 linear compounds have been reported as byproducts of PE degradation (Hakkarainen and Albertsson 2004). These molecules span a broad range of sizes and structures (C1–C30) and encompass diverse physicochemical properties. They include hydrophobic hydrocarbons such as alkanes and alkenes, as well as a wide range of oxidised derivatives, including alcohols, aldehydes, ketones, carboxylic acids and esters.

Valorisation strategies based on chemical catalysis and microbial conversion have shown promise for repurposing some of these derivatives. To date, microbial studies have predominantly focused on the highly oxidised fraction of PE degradation products, particularly mono‐ and dicarboxylic acids, whose relatively high solubility and direct overlap with fatty acid catabolism facilitate their assimilation (Werner et al. 2021). In contrast, upstream oxidation intermediates, such as long‐chain alcohols, remain poorly understood. Their higher hydrophobicity and limited solubility likely constrain bioavailability and metabolic processing. These constraints potentially create a major bottleneck in the biological valorisation of polyethylene‐derived compounds. As a result, biotechnological strategies targeting these poorly soluble intermediates remain largely unexplored.

*Pseudomonas putida* KT2440, a bacterium renowned for its metabolic versatility, robustness and genetic tractability, has emerged as a leading platform for biotechnological applications (Weimer et al. 2020; Martínez‐García et al. 2014; Nikel and de Lorenzo 2018). KT2440 has also been explored for the bioconversion of plastic‐derived compounds, where polymer depolymerisation products can be assimilated into biomass and value‐added chemicals. For example, engineered KT2440 strains can metabolise the polyethylene terephthalate (PET) monomers, ethylene glycol and terephthalic acid (Freund et al. 2025). Among polyethylene‐derived compounds, KT2440 can natively metabolise oxidised intermediates, such as mono‐ and dicarboxylic acids with chain lengths ranging from 4 to 22 carbon atoms (Sullivan et al. 2022). Moreover, KT2440 is able to utilise 1‐alcohols as carbon sources, including short‐ (e.g., ethanol, C2OH) and medium‐chain (e.g., 1‐dodecanol, C12OH) (Thompson et al. 2020; Lu et al. 2023). If KT2440 can use larger alcohols is not yet established.

Here, we examined *P. putida* KT2440's ability to grow on fatty alcohols, 1‐hexadecanol (C16OH) and 1‐eicosanol (C20OH), as carbon sources. We found that it can utilise these molecules as a sole carbon and energy source, thereby expanding the known repertoire of recalcitrant molecules that this bacterium already metabolises. Furthermore, we performed five cycles of adaptive laboratory evolution (ALE) to generate variants with enhanced capacity to utilise these molecules. In this short time, the isolated adapted variants grew 1.4 (±0.1) times faster than the wild‐type strain. Comparative whole‐genome analysis of wild‐type versus adapted variants, along with experimental validation, revealed that substrate uptake, rather than metabolic processing itself, constitutes the primary bottleneck for the efficient utilisation of fatty alcohols. This work presents the fundamental understanding of fatty alcohol metabolism in *P. putida* KT2440 and establishes a foundation for engineering microbial platforms capable of valorising PE waste derivatives into valuable products, contributing to circular economy initiatives.

## 2 Experimental Procedures

### 2.1 Chemicals and Culture Media

All chemicals, including 1‐hexadecanol and 1‐eicosanol, as well as media components such as salts and trace elements, were purchased from Sigma‐Aldrich (St. Louis, MO, USA). All reagents were of analytical grade and used without further purification. LB was obtained commercially (Difco). AB minimal media was prepared with (NH_4_)_2_SO_4_ (2.0 g/L), Na_2_HPO_4_ (6.0 g/L), KH_2_PO_4_ (3.0 g/L), NaCl (3.0 g/L), and trace metals, CaCl_2_ (0.1 mM), MgCl_2_ (1.0 mM) and FeCl_3_ (3.0 μM). All experiments were performed using media sterilised by standard autoclaving.

### 2.2 Strains and Plasmids

All strains and plasmids used in this study are listed in Tables 1 and 2, respectively.

**TABLE 1:** Strains used in this work.

Name	Description	References
KT2440	*wild‐type* *Pseudomonas putida* KT2440 strain	Bagdasarian et al. (1981)
C16OH‐1	1‐hexadecanol ALE‐derived *P. putida* KT2440 variant 1	This work
C16OH‐2	1‐hexadecanol ALE‐derived *P. putida* KT2440 variant 2	This work
C20OH‐1	1‐eicosanol ALE‐derived *P. putida* KT2440 variant 1	This work
C20OH‐2	1‐eicosanol ALE‐derived *P. putida* KT2440 variant 2	This work
C20OH‐3	1‐eicosanol ALE‐derived *P. putida* KT2440 variant 3	This work
pGFP	*P. putida* KT2440 harbouring plasmid pS2313M	This work
pAlkL	*P. putida* KT2440 harbouring plasmid pS2313_AlkL	This work
DH5α	*Escherichia coli* cloning host: *F* ^*−*^ *𝜆* ^*−*^ *endA1 glnX44(AS) thiE1 recA1 relA1 spoT1 gyrA96(NaI* ^*R*^ *) rfbC1 feoR nupG ⲫ80(ΔlacZM15) Δ(argF‐lac)U169 hsdR17 (r*_*K*_^*−*^ *m*_*K*_^*+*^ *)*	Hanahan and Meselson (1983)
S17‐1	*Escherichia coli* conjugation donor strain: thi pro hsdR *recA* RP4‐2‐Tc::Mu‐Km::Tn7 integrated into chromosome	Simon et al. (1983)

**TABLE 2:** Plasmids used in this work.

Name	Description	References
pS2313M	Km^R^, *ori pBBR1*, *P*_*EM7*_, *msfgfp*	Volke et al. (2020)
pS2313_AlkL	Km^R^, *ori pBBR1*, *P*_*EM7*_, *alkL*	This work
pDONRPEX18Gm	Suicide vector: Gm^R^, *sacB* ^+^, ori_TRP4_, Gateway‐compatible	Hmelo et al. (2015)

### 2.3 Growth Experiments

*Pseudomonas putida* KT2440 was grown overnight on LB agar plates at 30°C. Three individual colonies were picked and used to inoculate 4 mL of liquid LB medium, which was incubated overnight at 30°C with shaking at 180 rpm. The resulting cultures were washed three times with AB minimal medium by centrifugation at 4000 rpm for 4 min. Cell pellets were resuspended in AB minimal medium without a carbon source and adjusted to an optical density (OD_600_) of 1.0. These cell suspensions were then used to inoculate 4 mL of AB medium supplemented with the corresponding carbon source at OD_600_ of 0.05. Growth was monitored by OD_600_ using a WPA CO 8000 Cell Density Meter (Biochrom Ltd. UK).

### 2.4 Adaptive Laboratory Evolution

Wild‐type *Pseudomonas putida* KT2440 was grown overnight on LB agar plates at 30°C. Three colonies were selected and grown overnight on 4 mL of LB media at 30°C and 200 rpm. These cultures were then washed with AB minimal media and inoculated into 12‐mL sterile glass tubes containing 4.0 mL of minimal media with 2.5 mM of 1‐hexadecanol or 1‐eicosanol as the sole carbon source and incubated at an initial OD_600_ of 0.05, 30°C, 180 rpm. Adaptive Laboratory Evolution (ALE) was performed by transferring an aliquot of the previous culture into a sterile tube containing freshly prepared AB minimal media with the same carbon source concentration to an initial OD_600_ of 0.05. All transfers were made when the population reached the maximum optical density for at least two consecutive days. This process was repeated until the growth rate was stabilised for three consecutive passages. A glycerol (30% v/v) stock was created for each population at the end of the ALE experiment, which was stored at −80°C.

### 2.5 Selection of Adapted Variants

An LB agar plate was streaked from every final ALE population and incubated overnight at 30°C. For each plate, three isolated colonies were randomly selected and grown overnight in 4‐mL LB media, resulting in a total of 36 isolates. The next day, a glycerol stock was created and stored at −80°C. Additionally, these same glycerol stocks were used to streak fresh LB agar plates. These last plates belong to a single isolate that was used for the adapted variant selection. Three biological replicates from each isolate were tested in minimal media with the corresponding carbon source. Growth was followed by measuring optical density (OD_600_) every 24 h. A single isolate from each population was selected for further analysis, giving rise to two adapted variants for C16OH and three for C20OH. The selection was based on growth rate and maximum optical density calculation based on custom python scripts. Finally, the optimised variants were subjected to whole‐genome sequence analysis.

### 2.6 Genetic Engineering

DNA fragments were amplified using standard PCR techniques with Q5 High‐Fidelity DNA Polymerase (New England Biolabs), following the manufacturer's instructions. PCR products were verified by size on 1% agarose gels stained with ethidium bromide. Oligonucleotide primers were synthesised by Sigma‐Aldrich (Israel) (Table S5). All plasmids were assembled using the NEBuilder HiFi DNA Assembly system (New England Biolabs).

All assembly reactions were transformed into *Escherichia coli* DH5α by electroporation (2.5 kV, 4–5 ms) using a MicroPulser device (Bio‐Rad Laboratories). Cells were recovered in 900 μL of LB medium and incubated for 2 h at 37°C with shaking (200 rpm), followed by selection on LB agar supplemented with kanamycin (50 μg/mL) or gentamicin (10 μg/mL). All plasmid constructs were verified by whole‐plasmid long‐read sequencing (Plasmidsaurus).

#### 2.6.1 AlkL Overexpression

The *alkL* gene, encoding the medium‐chain alkane transporter from *Pseudomonas putida* GPo1, was chemically synthesised and cloned into pET28a(+) by Twist Bioscience. This construct was used as template DNA to amplify *alkL* using primers ‘AlkL_for’ and ‘AlkL_rev’. The pS2313 backbone was amplified from pS2313M using primers ‘pS2313M_for’ and ‘pS2313M_rev’. Both fragments were assembled into a single construct. The resulting plasmid, pS2313_AlkL, is a broad‐host‐range vector carrying a kanamycin resistance cassette (KmR) and the pBBR1 origin of replication, with *alkL* expressed under the control of the constitutive synthetic promoter PEM7. The plasmid pS2313M, harbouring monomeric superfolder GFP (*msfGFP*), was used as a control in overexpression experiments.

For *P. putida* KT2440 WT transformation, a single colony was picked from a previously inoculated LB agar plate and grown overnight in a four millilitre LB culture at 30°C and 200 rpm. The cell pellet was recovered by centrifugation for 4 min and 4000 rpm at room temperature. Then, it was washed twice with ice‐cold, sterile 300 mM sucrose. The cell pellet was resuspended on 100 μL of the same solution. Such volume was mixed with 100 ng of either pS2313_AlkL or pS2313M and transferred to a sterile 1 mm electroporation cuvette. Plasmid DNA was incorporated by electroporation (25 kV, 4–5 ns) using a MicroPulser device (Bio‐Rad Laboratories). Cell suspension was recovered with 900 μL of LB and incubated for 2 h at 30°C and 200 rpm. Selection of transformant colonies was made by plating and incubating overnight on LB agar plates supplemented with kanamycin (50 μg/mL) at 30°C.

#### 2.6.2 Alcohol Dehydrogenases Deletions

Gene deletions were performed using a two‐step allelic exchange method based on homologous recombination (Hmelo et al. 2015). Non‐replicative deletion plasmids were constructed by amplifying ~500 bp regions upstream and downstream of each target gene from *Pseudomonas putida* KT2440 genomic DNA and cloning these flanking fragments into the pDONRPEX18Gm vector. For deletion of PP_5121/PP_5122, flanking regions were amplified using primers ds_PP_5121/22_for, ds_PP_5121/22_rev, ups_PP_5121/22_for and ups_PP_5121/22_rev. For deletion of PP_4760, flanking regions were amplified using primers ds_4760_for, ds_4760_rev, ups_4760_for and ups_4760_rev. The plasmid backbone was amplified using primers pDONR_for and pDONR_rev. All constructs were assembled as described above.

The resulting and sequenced pDONRPEX18Gm constructs were introduced into *P. putida* KT2440 derivatives via conjugation with *E. coli* S17‐1. Selection for the first homologous recombination event was performed on gentamicin media, resulting in chromosomal integration of the plasmid. Counterselection on sucrose‐containing plates enabled the identification of double crossover events, leading to the deletion of the target gene. Candidate mutants were screened and confirmed by PCR.

### 2.7 DNA Extraction and Whole‐Genome Sequence Analysis

The selected optimised variants were grown overnight in a 4‐mL LB culture grown at 30°C and 200 rpm. DNA was extracted using the *GenElute Bacterial Genomic DNA Kit* (Sigma‐Aldrich). DNA sequencing was performed using both long‐ and short‐read technologies. Long‐read sequencing (Oxford Nanopore) was carried out by Plasmidsaurus, while short‐read sequencing (Illumina) was performed by SeqCenter (Pittsburgh, PA, USA). All evolved variants were analysed using this combined sequencing approach. Raw sequencing reads were quality‐checked with FastQC and trimmed using Cutadapt (Martin 2011). The resulting reads were aligned against the lab reference *P. putida* KT2440 WT to detect genetic variants via *breseq* and *snippy*. The affected genes were handled as indicated in the following section.

### 2.8 Enrichment Analysis for Functional Categories

The reference genome was annotated using EggNOG‐mapper, to assign functional categories (Cantalapiedra et al. 2021). We then evaluated whether affected genes were enriched or depleted in specific clusters of orthologous genes (COG) categories relative to the genome‐wide background. For each COG category, we built a 2 × 2 contingency table that contrasts affected versus non‐affected genes (i.e., the remainder of the annotated genome after removing affected genes), ensuring the two groups are disjoint. The odds ratio was computed with a Haldane–Anscombe correction of +0.5 to all cells when zeros occurred, and two‐sided *p* values were obtained from the hypergeometric tail probabilities (Fisher's exact test). We excluded the *Unassigned* category from inferential testing but reported its counts for completeness; including it in the test is possible and does not affect the construction of the table. *p* values across categories were adjusted for multiple comparisons using the Benjamini–Hochberg procedure.

## 3 Results

### 3.1 *Pseudomonas putida* KT2440 Can Utilise Fatty Alcohols as a Sole Carbon Source

**FIGURE 1:**
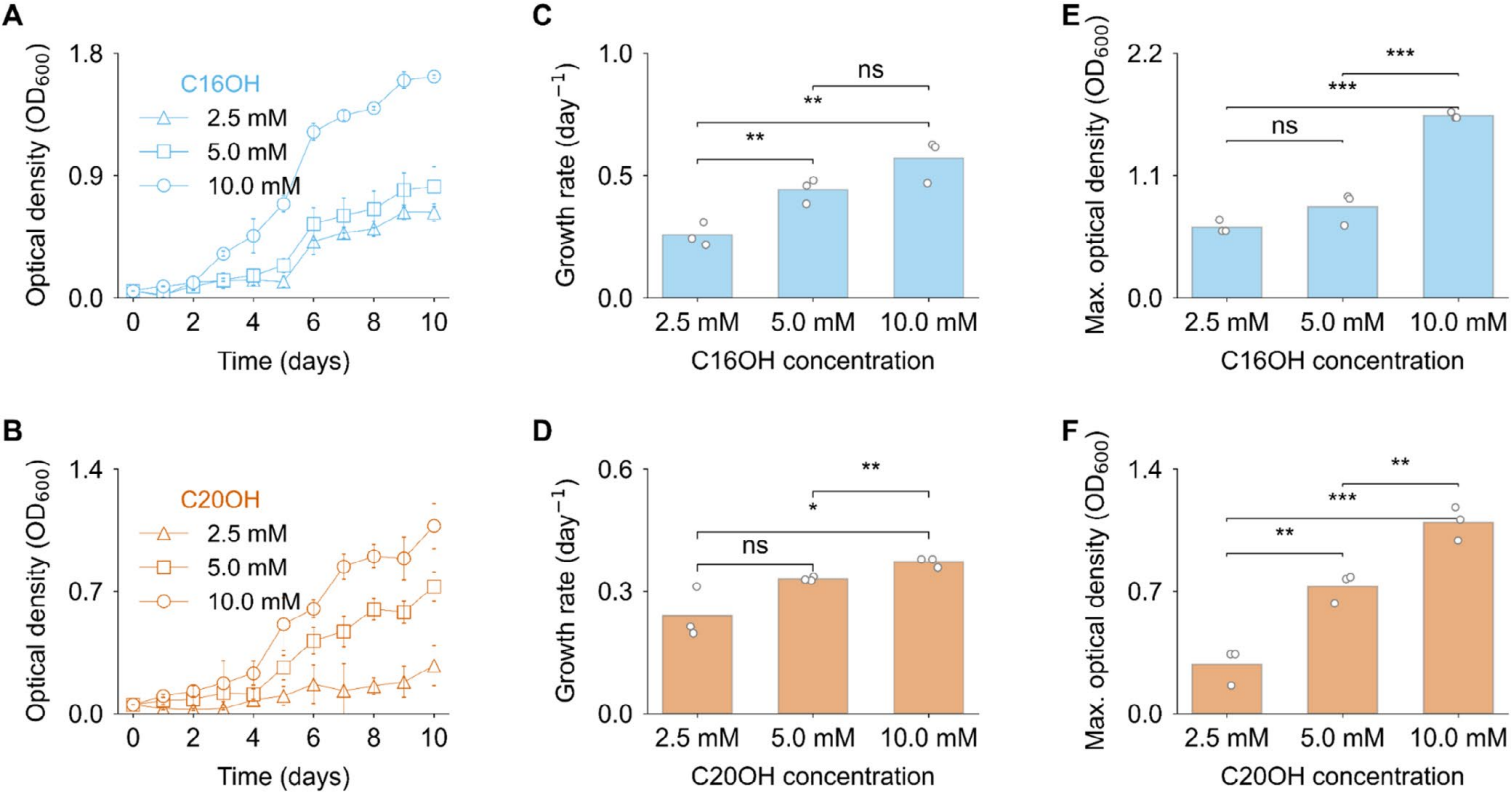
*Pseudomonas putida* KT2440 uses long‐chain alcohols as the sole carbon source. Growth curves of wild‐type *P. putida* KT2440 on varying concentrations of C16OH (A) and C20OH (B). Growth rate (C, D) and maximum optical density (E, F) for the tested concentrations. Error bars represent the standard deviation of three biological replicates. Individual data points are shown. Statistical significance was assessed using *t*‐tests; *p* < 0.05 (*), *p* < 0.01 (**), and not significant (ns). For visualisation purposes, growth curves are shown for the initial 10 days, although cultures were monitored until stationary phase (up to 23–25 days).

The utilisation of 1‐alcohols by wild‐type *Pseudomonas putida* KT2440 has been described only for short‐ and medium‐chain alcohols up to 12 carbon atoms in length (e.g., ethanol, C2OH to 1‐dodecanol, C12OH) (Thompson et al. 2020; Lu et al. 2023). Given the limited knowledge of its capacity to metabolise larger molecules, we tested whether wild‐type *P. putida* KT2440 could utilise 1‐hexadecanol (C16OH) and 1‐eicosanol (C20OH) as the sole carbon source. We tested three concentrations (2.5, 5.0 and 10.0 mM) to assess concentration‐dependent effects on growth (Figure 1A,B). These concentrations exceed the estimated aqueous solubility limits of both C16OH (~0.0002–0.004 mM) and C20OH (~0.000003–0.00003 mM). Growth rates and maximum optical density exhibited a clear dependence on substrate concentration. Higher concentrations support the fastest growth rates and maximum optical density for both fatty alcohols (Figure 1C–F). These findings indicate that the solubility of fatty alcohols does not appear to be a bottleneck for utilisation. Moreover, fatty alcohols do not show a concentration‐dependent toxicity, as reported with other aliphatic alcohol substrates such as 1‐octanol (Huesemann et al. 2004; Kongpol et al. 2014).

### 3.2 Adaptive Laboratory Evolution Enhances Wild‐Type *Pseudomonas putida* KT2440 Fatty Alcohols Utilisation

**FIGURE 2:**
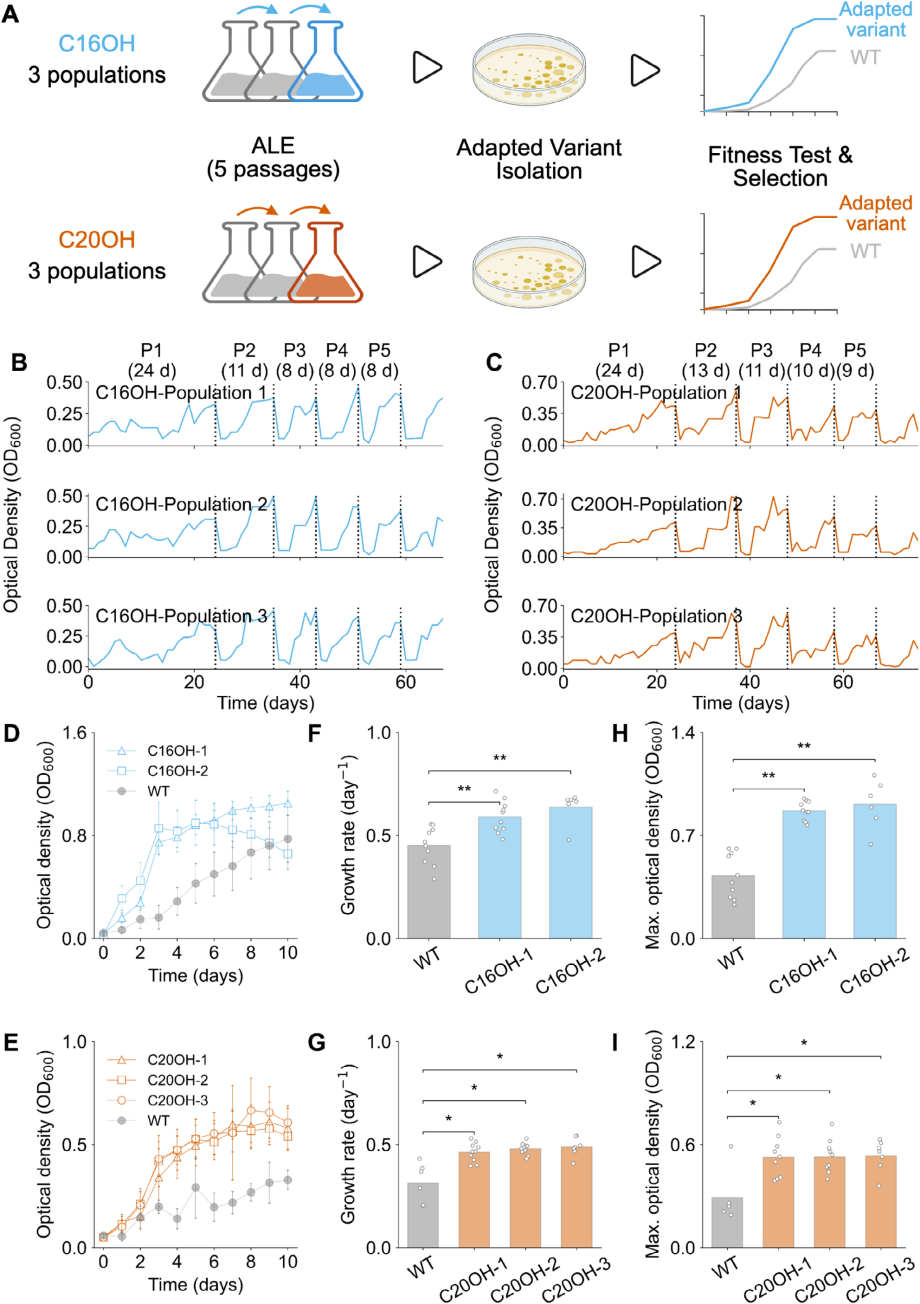
Adaptive laboratory evolution (ALE) of *Pseudomonas putida* KT2440 on long‐chain alcohol enhances the growth rate of adapted variants. (A) Schematic overview of the ALE workflow. Growth curves show optical density (OD_600_) trajectories for three independent populations of *Pseudomonas putida* KT2440 evolved on C16OH (B) and C20OH (C) across five serial passages. Vertical dashed lines indicate the time points at which cultures were transferred to fresh medium. The duration in days (d) of each passage (P) is indicated above. Growth curves of adapted variants in C16OH (D) and C20OH (E). Growth rate (day^−1^) in C16OH (F) and C20OH (G), and maximum optical density (OD_600_) in C16OH (H) and C20OH (I) of adapted variants and WT. Bars represent mean values; dots represent individual six to ten biological replicates. Statistical comparisons were performed using unpaired two‐sided *t*‐tests. Stars indicate significance levels: *p* < 0.05 (*), *p* < 0.01 (**). For visualisation purposes, growth curves are shown for the initial 10 days, although cultures were monitored until stationary phase (up to 23–25 days).

Having established that *P. putida* KT2440 can use fatty alcohols as a sole carbon and energy source, we sought to identify the bottleneck in their utilisation to guide future engineering (Sandberg et al. 2019). We performed five passages of adaptive laboratory evolution (ALE) using 1‐hexadecanol (C16OH, 2.5 mM) and 1‐eicosanol (C20OH, 2.5 mM) as the sole carbon and energy source (Figure 2A). We initiated three independent evolution lines for each substrate starting from wild‐type *P. putida* KT2440. Initially, we grew the wild‐type strain for 23 days in C16OH and for 25 days in C20OH. Cultures were then diluted into fresh minimal media to an initial optical density (OD_600_) of 0.05, containing the same carbon and energy source and maintaining the same concentration. For the next four passages, this dilution was performed when the OD_600_ remained unchanged over two consecutive days, indicating that the culture had entered the stationary phase. We observed homogeneous phenotypic optimisation immediately after the first passage, which remained consistent across the subsequent four passages. After passage one, dilution times decreased from 24 days to 11 days in C16OH and 13 days in C20OH and remained constant throughout the rest of the ALE process (Figure 2B,C). Across five passages, populations accumulated an estimated 14.1–14.5 generations on C16OH and 15.1–15.8 generations on C20OH.

At the end of the ALE, we randomly selected six isolates from each of the three populations using the two carbon sources (36 colonies in total). We tested their ability to utilise C16OH and C20OH in growth experiments using the same carbon source and same concentration (2.5 mM) from which they were obtained via ALE (Figure S1). From there, we selected the five best‐performing adapted variants, C16OH‐1 and C16OH‐2 from C16OH, and C20OH‐1, C20OH‐2 and C20OH‐3 from C20OH, and re‐tested their phenotypes (Figure 2D,E). The growth rate and maximum optical density show that the five adapted variants grow significantly faster than the wild‐type (1.35*x* ± 0.05 higher on C16OH, *p* < 0.05, and 1.4*x* ± 0.02 higher on C20OH, *p* < 0.05) (Figure 2F–I). Additionally, the specific growth rates of the adapted variants fell within the magnitude of the population from where they were isolated, indicating that the adapted variants well represent the population phenotype (Table S1).

### 3.3 Mutation Analysis Sheds Light on the Molecular Mechanisms Behind Optimised Growth in Fatty Alcohols

To elucidate the genetic basis underlying the improved growth phenotypes, we performed whole‐genome sequencing on the wild‐type strain and the five adapted variants. To obtain a high‐quality ancestral genome and facilitate the identification of plausible adaptive mutations in our isolated adapted variants, we re‐sequenced the parental wild‐type strain using a hybrid long‐ and short‐read approach. The genome was assembled, annotated and compared to the reference genome deposited on the NCBI repository (*GenBank*: AE015451.2) (Belda et al. 2016). Our wild‐type genome and the reference genome differ only by 22 single‐nucleotide polymorphisms (SNPs) and 27 short insertions or deletions (indels) consistent with strain variation (Table S2) (Freddolino et al. 2012). The genomes of the adapted variants were also obtained via long and short‐read sequencing and compared with our wild‐type genome. Mutation calling was performed using *breseq* (Barrick et al. 2014) and *snippy* (Seemann 2015), both of which show the same mutation list. Long reads were assembled and compared against the parental strain genome, revealing no genomic rearrangements.

Two adapted variants, C20OH‐2 and C20OH‐3, contained six and seven mutations, respectively. In contrast, C16OH‐1, C16OH‐2 and C20OH‐1 each carried 30 to 38 mutations. These variants shared the same non‐synonymous SNP, V466E (GTG to GAG) in the *mutL* gene, which encodes the DNA mismatch repair endonuclease, MutL (Modrich 2016). In bacteria, MutL residues around 467–479 fall in the C‐terminal endonuclease/metal‐binding region that harbours the conserved DQHA(X)_2_E(X)_2_E motif required for MutL's nicking activity. Alterations in this motif often attenuate mismatch repair and elevate mutation rates (i.e., hypermutation phenotype) as observed here (Correa et al. 2011).

Including those of the hypermutants, we detected a total of 91 mutations across the five adapted variants, including 63 SNPs and 28 short indels. Of these, 77 were located in coding regions and 14 in intergenic areas. Of the 77 mutations in coding regions, 71 are non‐synonymous mutations. These were located in 47 unique genes, indicating some levels of mutational convergence (Table S3).

**FIGURE 3:**
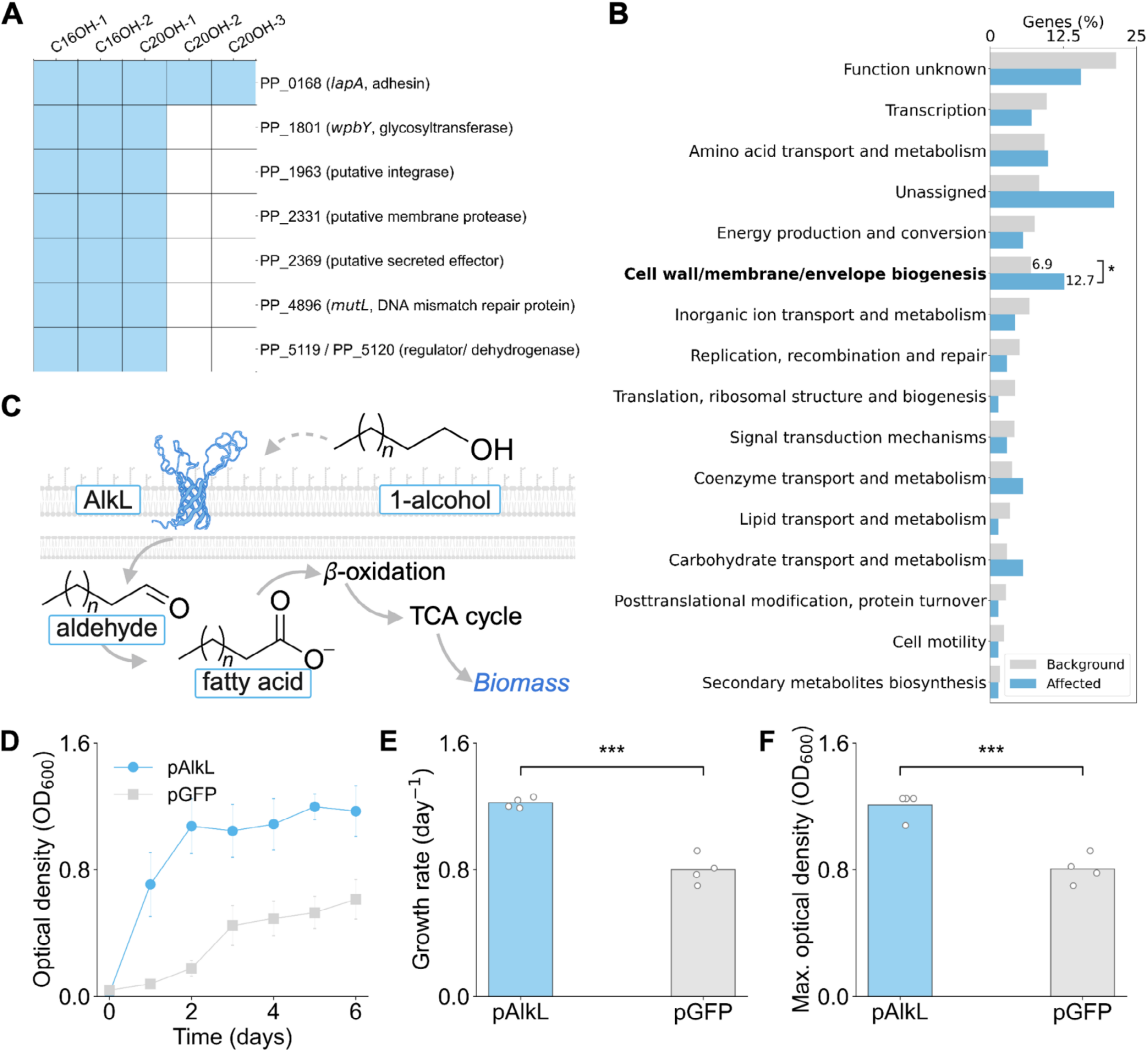
Mutational analysis points to a membrane‐related adaptation mechanism. (A) Heatmap showing the occurrence of convergent non‐synonymous mutations in C16OH and C20OH adapted variants. (B) Bar plot derived from the enrichment analysis by functional categories. COG categories which yielded zero occurrences in the affected genes were omitted for visualisation purposes. The star (*) indicates a significance level: *q* < 0.05. (C) Schematic representation of AlkL‐mediated transport, highlighting its outer membrane localisation and proposed role in facilitating interaction with hydrophobic substrates such as long‐chain alcohols. (D) Growth of wild‐type *Pseudomonas putida* KT2440 expressing the heterologous AlkL transporter (pAlkL) or GFP as control (pGFP) on C16OH. Growth rate (E) and maximum optical density (F) for the engineered strains. Bars represent the mean value of four biological replicates, with individual data points overlaid. Stars indicate significance levels: *p* < 0.01 (***). For visualisation purposes, growth curves are shown for the initial 10 days, although cultures were monitored until stationary phase (up to 23–25 days).

To dissect the plausible contribution of mutations to the phenotype, we analysed convergent mutations at the gene level across independent replicas, excluding synonymous mutations. Such analysis often highlights mutations that confer advantageous functions under the selection conditions of the experiment (Zhu et al. 2018; Mohamed et al. 2020; Ackermann et al. 2021). We found six mutations converging in the two C16OH‐adapted variants, and two converging across the three C20OH‐adapted variants (Table S4). Regardless of the carbon source from which they evolved, we identified eight convergent mutations (identical base) in at least three of the five isolates (Figure 3A, Table S4). Beyond convergence, we also analysed the function of each mutated gene individually. The next sections will describe the hypotheses and demonstrations we developed from these analyses.

#### 3.3.1 Mutations in Alcohol Dehydrogenases Are Not Essential to Growth Improvement

From the convergence analysis, we observed that the adapted variants C16OH‐1, C16OH‐2 and C20OH‐1 harbour an SNP (G to A) in the intergenic region between genes PP_5119 (transcriptional regulator) and PP_5120 (aldehyde dehydrogenase). PP_5119 and PP_5120 are upstream of the genes PP_5121 and PP_5122, forming what appears to be a small operon (Figure S2A). Thus, we hypothesised that a mutation upstream of the transcriptional regulator (PP_5119) could affect the operon's overall transcriptional level, revealing a functional role for these genes in the fatty alcohol metabolism of *P. putida* KT2440. LaoA and LaoB are the two subunits of an alcohol dehydrogenase, essential for growth in fatty alcohols ranging from 12 to 24 carbons (C12OH to C24OH) (Panasia and Philipp 2018). This is of particular interest because the genes PP_5121 and PP_5122 are homologous to the gene products of *laoB* (55% sequence identity) and *laoA* (70% sequence identity) in *Pseudomonas aeruginosa* PAO1, respectively.

We tested whether PP_5121 and PP_5122, homologues of *laoA* and *laoB*, are required for growth on long‐chain alcohols in *Pseudomonas putida* KT2440. To do so, we evaluated growth of both the wild‐type strain and the adapted variants (C16OH‐1 and C20OH‐1) carrying deletions of PP_5121/PP_5122 across different substrate concentrations. In the wild‐type background, deletion of PP_5121/PP_5122 did not impair growth on C16OH or C20OH at either 2.5 mM or 10 mM (Figure S2B,C,E,F). Similarly, deletion of these genes in the adapted variants (C16OH‐1ΔPP_5121&2 and C20OH‐1ΔPP_5121&2) did not affect their improved growth phenotype (Figure S2D,G). These results indicate that the LaoA and LaoB homologues are not essential for fatty alcohol utilisation under the tested conditions.

Given that alcohol dehydrogenases are expected to catalyse the initial oxidation step, we further examined additional candidates. We identified a SNP (G to A) located 93 bp upstream of PP_4760, a predicted alcohol dehydrogenase, in the adapted variant C16OH‐1. To assess its contribution, we generated deletions of PP_4760 in both the wild‐type and adapted backgrounds. As with PP_5121/PP_5122, deletion of PP_4760 did not impair growth on C16OH or C20OH at 2.5 mM or 10 mM in the wild‐type strain (Figure S2B,C,E,F), nor did it affect the phenotype of the adapted variants (Figure S2D,G).

Importantly, no additional mutations were identified in previously characterised alcohol dehydrogenases, including *adhP* (PP_3839), *pedE* (PP_2674) and *pedH* (PP_2679) or their intergenic regions, which are known to mediate oxidation of short‐ and medium‐chain alcohols (Thompson et al. 2020). Together, these results indicate that PP_5121, PP_5122 and PP_4760 are not individually required for long‐chain alcohol metabolism. Given that the *P. putida* KT2440 genome encodes at least 14 putative alcohol dehydrogenases, our data support a model in which alcohol oxidation is metabolically robust and not a limiting step under the tested conditions.

#### 3.3.2 Substrate Uptake Is the Main Bottleneck in Fatty Alcohol Metabolism

We extended our analysis beyond catabolic genes to include all remaining mutations. In the convergence analysis, we observed four non‐synonymous convergent changes occurring in membrane–associated genes across the adapted variants. These included: (1) *lapA* (PP_0168), mutated in all five variants, which encodes a surface adhesin, (2) *wpbY* (PP_1801), detected in three variants, encoding a structural homologue of WaaG, a glycosyltransferase implicated in lipopolysaccharide biosynthesis in *P. aeruginosa* , (3) PP_2331, present in three variants, predicted to encode a membrane protease, and (4) PP_2369, also identified in three variants, annotated as a putative type III secreted effector‐like protein (Scaletti et al. 2023; Espinosa‐Urgel and Ramos‐González 2023).

The recurrent mutation, Q3355N, in LapA (PP_0168) maps to the central region of the protein, which is implicated in modulating adhesive properties. A functional study of the LapA homologue in *P. fluorescens* Ff0‐1 has shown that alterations in the central repeat domains significantly affect surface attachment and biofilm formation (Boyd et al. 2014). In addition, a single‐base deletion within a homopolymeric G tract (G_5_ to G_4_) in the PP_1801 coding sequence results in a frameshift from codon 304 and a premature stop at codon 317, truncating the final 64 amino acids of the protein. Based on sequence identity, PP_1801 is homologous to the *P. aeruginosa* glycosyltransferase, WaaG, whose C‐terminal region contributes to the sugar recognition; this truncation is expected to strongly impair enzyme function and alter cell‐surface polysaccharide architecture (Scaletti et al. 2023). Together, mutations affecting lipopolysaccharide biosynthesis and other membrane‐associated proteins, this pattern suggests selection for altered hypothetical cell–surface interactions. Although these loci have distinct primary functions, they collectively point to adaptive remodelling of the cell envelope. Such modifications could enhance adhesion to hydrophobic substrates, such as long‐chain alcohols, by increasing effective cell–substrate contact and improving substrate accessibility.

*P. putida* KT2440's genome encodes 545 transporter genes (approximately 10% of its proteome). It is known that transporters can be promiscuous or multifunctional (Lewinson et al. 2006). Thus, it is tempting to speculate that different transporters could be mutated in different strains to achieve the same phenotype optimisation. This genomic robustness may obscure clear patterns of gene‐level convergence even when the phenotypic outcome is similar. This was indeed the case when we manually analysed mutations regardless of convergence. For example, the isolate C16OH‐1 exhibits non‐synonymous mutations in PP_2656, a homologue of *ptsS* involved in cell adhesion in *P. aeruginosa* PAO1, and in PP_0880, which encodes an ABC transporter permease (Neznansky et al. 2014). C16OH‐2 also harbours mutations in membrane‐associated proteins, but different ones. We identified a non‐synonymous SNP in PP_2754, which encodes a putative outer membrane porin of the OprD family, crucial for nutrient uptake in *P. aeruginosa* PAO1 (Tamber et al. 2006).

To test whether adaptive mutations were preferentially found in envelope‐associated proteins, we performed a functional category enrichment analysis on the mutational targets. We compiled a set by including all unique genes with non‐synonymous coding mutations in all five adapted variants (47 genes). Also, if the mutation was found in an intergenic region, we included the two flanking genes to capture hypothetical regulatory effects on adjacent loci (26 genes). The total of 73 unique affected genes was assigned a single Clusters of Orthologous Groups (COG) category using eggNOG‐mapper (Cantalapiedra et al. 2021). We compared the obtained gene set to the occurrence of all COG‐assigned genes in wild‐type *P. putida* KT2440 using two‐sided Fisher's exact tests with Benjamini‐Hochberg correction (see *Experimental Procedures*). This analysis revealed a significant enrichment of the *cell wall/membrane/envelope biogenesis* COG category, consistent with envelope remodelling as a principal adaptive axis during growth on fatty alcohols (affected genes 12.7% vs. background genes 6.9%; Fisher's exact *q* < 0.05; Figure 3B).

We then hypothesised, based on our functional results, that substrate accessibility at the cell surface plays a central role in the utilisation of long‐chain alcohols. Specifically, (1) alcohol metabolism appears robust in KT2440, as knockout strains of alcohol dehydrogenases and their sequential oxidation pathways did not show a reduced phenotype, and (2) enrichment analysis points toward envelope‐associated functions. Together, these findings suggest that interactions at the cell surface, likely mediated by substrate accessibility and uptake, are key determinants for efficient long‐chain alcohol utilisation (Figure 3C). To experimentally validate whether improving cell–substrate interactions is important for fatty alcohol metabolism, we modified the wild‐type strain to express the *alkL* gene from *Pseudomonas putida* GPo1 (Figure 3C). AlkL is a characterised transporter that facilitates the import of C7–C16 n‐alkanes across the outer membrane (Grant et al. 2011, 2014). Previous studies have shown that it also facilitates the transport of medium‐chain alcohols (C8OH to C12OH) (Lu et al. 2023). Growth assays were performed by comparing wild‐type strains harbouring either the *alkL*‐ or *gfp*‐expressing plasmid. Remarkably, the expression of *alkL* significantly enhanced substrate utilisation as indicated by growth rate and maximum optical density on C16OH (Figure 3D–F). The improvements matched the phenotypic gains observed in the C16OH‐adapted variants, providing independent confirmation of the critical role of substrate uptake. As AlkL cannot accommodate substrates larger than C16, its overexpression did not enhance the growth of the wild‐type strain in C20OH (Grant et al. 2014).

## 4 Discussion

In this study, we demonstrate that wild‐type *Pseudomonas putida* KT2440 can utilise long‐chain aliphatic alcohols (fatty alcohols) such as 1‐hexadecanol (C16OH) and 1‐eicosanol (C20OH) as the sole carbon source. While previous reports described *P. putida* KT2440 growth only on short‐ and medium‐chain alcohols (up to C12OH) (Thompson et al. 2020; Lu et al. 2023), our results extend this substrate range to longer and less soluble alcohols relevant to the PE circular economy. Growth correlated strongly with substrate concentration, with high concentrations supporting faster growth rates and higher maximum optical density. This trend contrasts with that observed for medium‐chain alcohols, such as 1‐octanol (C8OH), where increased concentrations lead to toxicity rather than improved growth (Lu et al. 2023). This suggests that at longer chain lengths, toxicity is attenuated, likely due to reduced solubility, allowing *P. putida* KT2440 to exploit fatty alcohols when sufficient substrate is available in the media.

Five adaptive laboratory evolution passages on C16OH and C20OH revealed an adaptive response, with improvements in growth kinetics after the first passage and stable performance thereafter. These results are in line with previous ALE studies in *P. putida* KT2440, where early adaptive sweeps also quickly shaped evolving populations, making population‐level and clonal phenotypes very similar (Lim et al. 2021; Declerck et al. 2025). The fact that our adapted variants grew as fast as their parent populations suggests that beneficial mutations spread and reached fixation.

Through mutational analysis of the adapted variants compared with wildtype, we identified an intergenic mutation upstream of a small operon containing homologues of LaoAB, which are known to be essential for long‐chain alcohol metabolism in *P. aeruginosa* PAO1 (Panasia and Philipp 2018), and a putative alcohol dehydrogenase (PP_4760). Although alcohol dehydrogenases should be essential for growth on fatty alcohols, deleting these genes in the adapted variants showed no observable impact on growth (Panasia and Philipp 2018). *P. putida* KT2440 encodes at least 14 alcohol dehydrogenases compared to 10 in *P. aeruginosa* PAO1 (Thompson et al. 2020). Such redundancy likely provides robustness (Fox et al. 1992; Wehrmann et al. 2017). Indeed, our data suggest that alcohol‐oxidation capacity is not a limiting factor in fatty alcohol metabolism.

Moreover, mutations occurred consistently in genes involved in cell envelope and membrane functions. We identified four recurrent non‐synonymous changes in membrane‐associated genes. We also observed a significant enrichment of the *cell wall/membrane/envelope* biogenesis COG category among mutated genes, implicating envelope dynamics as a key adaptive axis during growth on fatty alcohols. Such mutations likely facilitated improved substrate access by altering surface hydrophobicity, permeability or adhesion to hydrophobic substrates (Blesken et al. 2020). To experimentally test whether improving cell–substrate interactions and substrate uptake could enhance fatty alcohol utilisation, we expressed AlkL from *P. putida* GPo1 in the wild‐type strain. AlkL is a characterised outer membrane transporter facilitating the import of medium‐chain alkanes and alcohols (Grant et al. 2011, 2014). Its expression significantly enhanced growth on C16OH but not on C20OH, consistent with the known alkane‐size limit of AlkL (up to C16). These results reinforce that substrate uptake capacity determines the upper limit of assimilable chain length, and that improved membrane permeability or transporter activity can directly enhance growth on fatty alcohols.

*P. putida* KT2440 encodes an unusually large repertoire of transporter genes (545 predicted, ~10% of its proteome) (Belda et al. 2016). To test whether any of these could act as an AlkL‐like alkane transporter, we used Foldseek (Van Kempen et al. 2024) to compare the structure of AlkL against predicted structures of all KT2440 proteins annotated as transporters. Despite this extensive repertoire, neither sequence nor structural similarity to known alkane transport systems was identified (Liu et al. 2022; Rao et al. 2025). This suggests that either uncharacterised transporters mediate fatty alcohol uptake or that membrane remodelling collectively compensates for the lack of dedicated transport mechanisms. Notably, similar constraints have been reported for other substrates in KT2440, where uptake rather than intracellular metabolism can limit growth, as shown for aromatic compounds and diols (Brandenberg et al. 2022; Wada et al. 2021).

## 5 Conclusions

Our study demonstrates that *Pseudomonas putida* KT2440 can utilise long‐chain alcohols, such as 1‐hexadecanol and 1‐eicosanol, as a carbon source. It can evolve to efficiently assimilate these molecules, overcoming barriers related to their poor solubility. Through adaptive laboratory evolution, we generated isolates with improved growth phenotypes, supported by stable, heritable genetic changes. Genomic and functional analyses revealed that mutations in membrane‐associated genes, including surface adhesion proteins and potential transporters, drove these adaptations. These findings suggest that enhancing cell–substrate interactions is a key adaptive strategy for overcoming physical limitations to substrate uptake. Engineering the parental strain to express the *alkL* transporter from *P. putida* GPo1 reproduced the growth improvements on C16OH, providing experimental validation of the membrane transport hypothesis. Together, our results highlight the evolutionary plasticity of *P. putida* KT2440 and identify membrane remodelling as a promising target for future metabolic engineering strategies to valorise recalcitrant hydrophobic substrates.

## Author Contributions

**Lianet Noda‐García:** conceptualization, methodology, supervision, funding acquisition, project administration, writing – original draft, writing – review and editing. **Raul Mireles:** conceptualization, methodology, data curation, investigation, formal analysis, writing – original draft, writing – review and editing.

## Funding

The authors have nothing to report.

## Conflicts of Interest

The authors declare no conflicts of interest.

## Supporting information


**Figure S1:** Isolated adapted variants show improved growth compared to wild‐type (WT).
**Figure S2:** Deletion of genes coding putative alcohol dehydrogenases does not impair growth on C16OH and C20OH.
**Table S1:** Growth rate for wild‐type *Pseudomonas putida* KT2440 in long‐chain alcohols (2.5, 5.0 and 10.0 mM).
**Table S2:** Mutations in the wild‐type strain compared to the NCBI reference genome. Arrows indicate the direction of transcription of each gene relative to the genome.
**Table S4:** Occurrence of synonymous and non‐synonymous convergent mutations in C16OH and C20OH variants.
**Table S5:** Primers used in this work.

**Table S3:** Mutations identified in adaptive laboratory evolution (ALE)‐derived KT2440 variants adapted for growth on long‐chain alcohols.

## Data Availability

The datasets generated and/or analysed during the current study are available from the corresponding author upon reasonable request.
