# Supplementary material for: Adaptive Laboratory Evolution Unlocks Membrane Permeability as a Key Limitation in Long‐Chain Alcohol Metabolism by Pseudomonas putida KT2440
Source: Microb Biotechnol. 2026 Jun 23;19(6):e70399. doi: 10.1111/1751-7915.70399 (PMC13291428; doi:10.1111/1751-7915.70399)
Supplement: Supplementary file 1 — Figure S1: Isolated adapted variants show improved growth compared to wild‐type (WT). Figure S2: Deletion of genes coding putative alcohol dehydrogenases does not impair growth on C16OH and C20OH. Table S1: Growth rate for wild‐type Pseudomonas putida KT2440 in long‐chain alcohols (2.5, 5.0 and 10.0 mM). Table S2: Mutations in the wild‐type strain compared to the NCBI reference genome. Arrows indicate the direction of transcription of each gene relative to the genome. Table S4: Occurrence of synonymous and non‐synonymous convergent mutations in C16OH and C20OH variants. Table S5: Primers used in this work. [file MBT2-19-e70399-s002.docx]

**Adaptive laboratory evolution unlocks membrane permeability as a key limitation in long-chain alcohol metabolism by *Pseudomonas putida* KT2440**

Raúl Mireles^a^ & Lianet Noda-García^a,*^

^a^ Department of Plant Pathology and Microbiology, Institute of Environmental Sciences, The Robert H. Smith Faculty of Agriculture, Food and Environment, The Hebrew University of Jerusalem. Rehovot, Israel.

* Corresponding author: Lianet Noda-García (lianet.noda@mail.huji.ac.il)

**Supplementary Information**

[Figure S1. Isolated adapted variants show improved growth compared to wild-type (WT). 2](#_heading=h.ccw1h61lmwxy)

[Figure S2. Deletion of genes coding putative alcohol dehydrogenases does not impair growth on C16OH and C20OH. 3](#_heading=h.flnnmdsgfwf8)

[Table S1. Growth rate for wild-type *Pseudomonas putida* KT2440 in long-chain alcohols (2.5, 5.0 and 10.0 mM). 4](#_heading=h.8mt94ixl2n9f)

[Table S2. Mutations in the wild-type strain compared to the NCBI reference genome. Arrows indicate the direction of transcription of each gene relative to the genome. 4](#_heading=h.q7drrc6oevad)

[Table S4. Occurrence of synonymous and non-synonymous convergent mutations in C16OH and C20OH variants. 7](#_heading=h.n51houita8l0)

[Table S5. Primers used in this work. 8](#_heading=h.r35lr1z5c4ud)


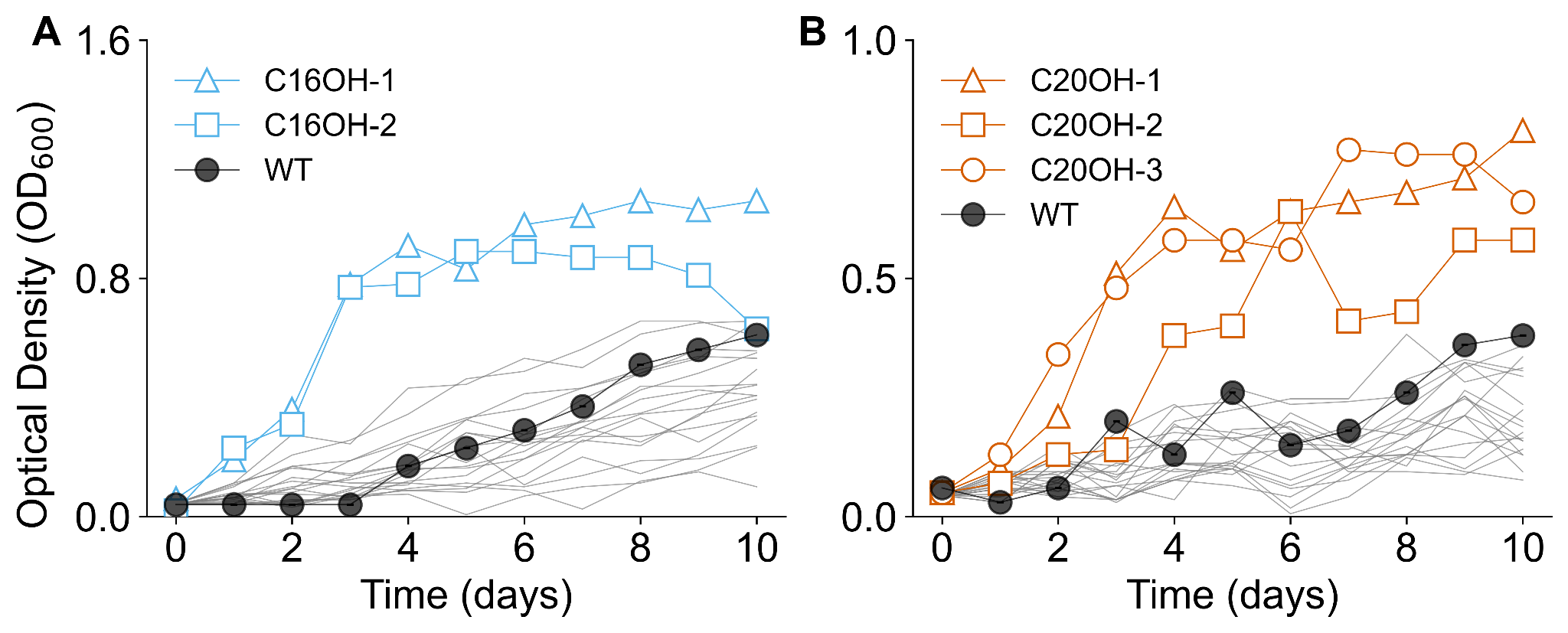


# **Figure S1. Isolated adapted variants show improved growth compared to *wild-type* (WT).**

Selected adapted variants in C16OH (A) and C20OH (B) show markedly improved growth relative to wild-type (WT, black solid circles), while screened non-selected isolates are shown in gray.

# **
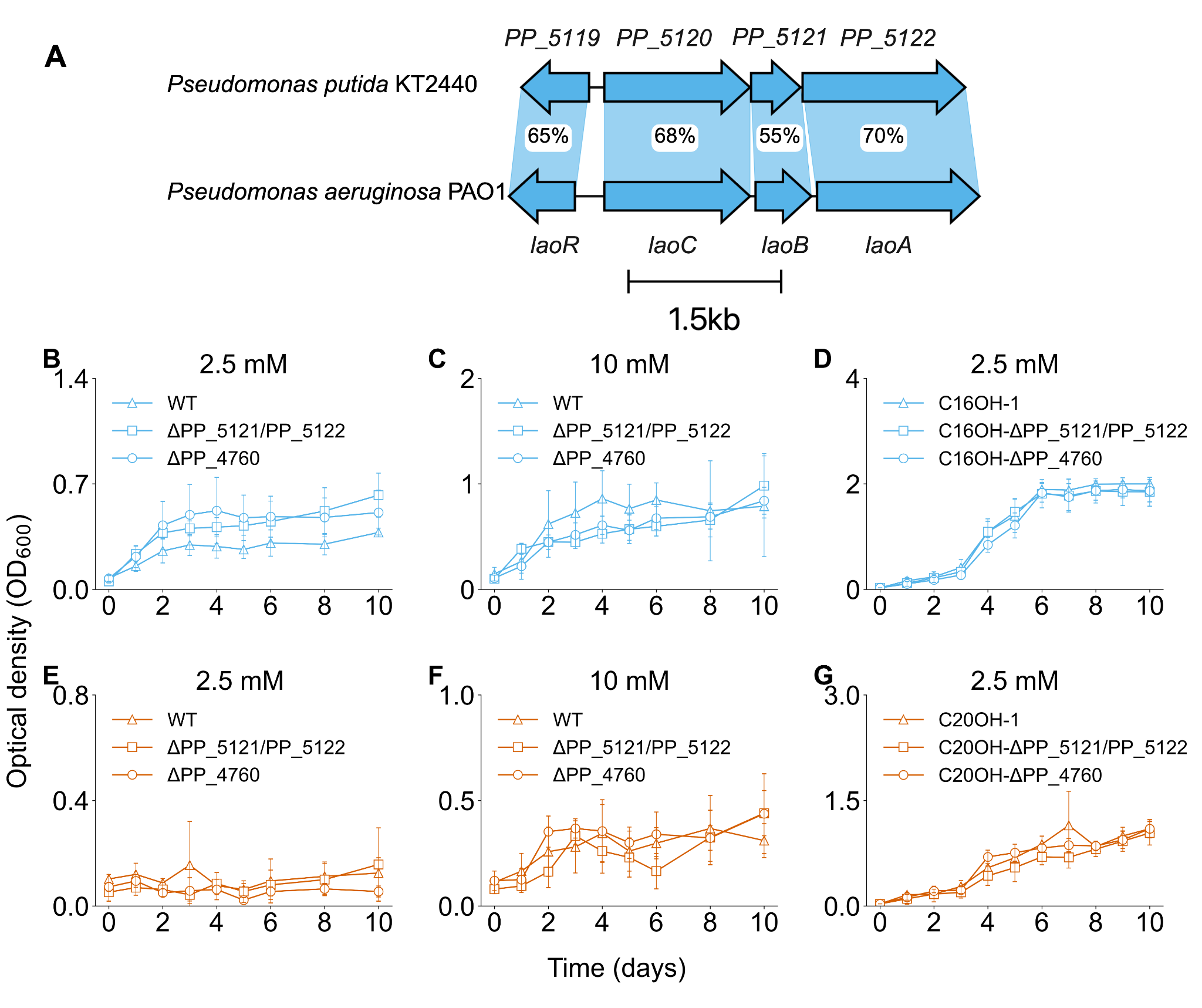
Figure S2. Deletion of genes coding putative alcohol dehydrogenases does not impair growth on C16OH and C20OH.**

(A) Genomic organization of the homologous laoABCR locus in *P. putida* KT2440 and *P. aeruginosa* PAO1. Arrows indicate the open reading frames and transcriptional orientation. Percentages denote amino-acid sequence identity. (B–D) Growth of wild-type (WT) and deletion strains (ΔPP_5121/PP_5122 and ΔPP_4760) on C16OH at 2.5 mM (B), 10 mM (C), and the adapted strain, C16OH-1, at 2.5 mM (D). Growth of wild-type (WT) and deletion strains (ΔPP_5121/PP_5122 and ΔPP_4760) on C20OH at 2.5 mM (E), 10 mM (F), the adapted strain, C20OH-1, at 2.5 mM (G).

# **Table S1. Growth rate for wild-type *Pseudomonas putida* KT2440 in long-chain alcohols (2.5, 5.0 and 10.0 mM).**

| Substrate | Concentration | | |
| --- | --- | --- | --- |
|  | 2.5 mM | 5.0 mM | 10.0 mM |
| C16OH | 0.23 ± 0.04 day^-1^ | 0.32 ± 0.10 day^-1^ | 0.55 ± 0.03 day^-1^ |
| C20OH | 0.03 ± 0.07 day^-1^ | 0.42 ± 0.02 day^-1^ | 0.37 ± 0.03 day^-1^ |

# **Table S2. Mutations in the wild-type strain compared to the NCBI reference genome.** Arrows indicate the direction of transcription of each gene relative to the genome.

| Mutation | Locus Tag | Annotation |
| --- | --- | --- |
| +A | *PP_0224* ← / ← *sctC* | Monooxygenase/ ABC transporter |
| +C | *hslO → / → PP_0254* | chaperonin/unknown function |
| +T | *PP_0278* → | unknown function |
| A→G | *hisF* → / ← *cbcV* | phosphate synthase/ABC transporter |
| A→G | *uhpA* → / → *PP_0411* | regulator UhpA/ABC transporter |
| +C | *tdcG‑II* ← / ← *gcvP‑I* | L‑serine dehydratase/glycine dehydrogenase |
| +C | *PP_1242* ← / ← *PP_1244* | unknown function/unknown function |
| 2 bp→AC | *trpS → / → zapE* | tRNA ligase/ nucleoside triphosphate hydrolase |
| 1 bp→CG | *trpS → / → zapE* | tRNA ligase/ nucleoside triphosphate hydrolase |
| +C | *trpS → / → zapE* | tRNA ligase/ nucleoside triphosphate hydrolase |
| +C | *rluA ← / ← minE* | rRNA and tRNA synthase/Cell division factor |
| C→T | *PP_2589 →* | Aldehyde dehydrogenase family protein |
| +G | *PP_3394 ← / ← PP_3395* | CoA lyase/Transcriptional regulator |
| T→C | *PP_3486 ← / ← PP_3487* | cytochrome c/unknown function |
| A→T | *PP_3486 ← / ← PP_3487* | cytochrome c/unknown function |
| 2 bp→TC | *PP_4061 → / ← PP_4063* | unknown function/‑CoA ligase |
| +G | *PP_4061 → / ← PP_4063* | unknown function/‑CoA ligase |
| +C | *PP_4061 → / ← PP_4063* | unknown function/‑CoA ligase |
| (T)_7→6_ | *gltA → / ← yeiW* | citrate synthase/putative oxidoreductase |
| T→G | *gltA → / ← yeiW* | citrate synthase/putative oxidoreductase |
| 2 bp→CT | *gltA → / ← yeiW* | citrate synthase/putative oxidoreductase |
| +C | *gltA → / ← yeiW* | citrate synthase/putative oxidoreductase |
| A→G | *gltA → / ← yeiW* | citrate synthase/putative oxidoreductase |
| C→T | *gltA → / ← yeiW* | citrate synthase/putative oxidoreductase |
| C→G | *gltA → / ← yeiW* | citrate synthase/putative oxidoreductase |
| 2 bp→CT | *gltA → / ← yeiW* | citrate synthase/putative oxidoreductase |
| Δ1 bp | *gltA → / ← yeiW* | citrate synthase/putative oxidoreductase |
| +T | *gltA → / ← yeiW* | citrate synthase/putative oxidoreductase |
| A→G | *gltA → / ← yeiW* | citrate synthase/putative oxidoreductase |
| A→C | *gltA → / ← yeiW* | citrate synthase/putative oxidoreductase |
| Δ1 bp | *gltA → / ← yeiW* | citrate synthase/putative oxidoreductase |
| A→G | *gltA → / ← yeiW* | citrate synthase/putative oxidoreductase |
| A→C | *gltA → / ← yeiW* | citrate synthase/putative oxidoreductase |
| G→C | *gltA → / ← yeiW* | citrate synthase/putative oxidoreductase |
| C→T | *gltA → / ← yeiW* | citrate synthase/putative oxidoreductase |
| +T | *gltA → / ← yeiW* | citrate synthase/putative oxidoreductase |
| C→G | *gltA → / ← yeiW* | citrate synthase/putative oxidoreductase |
| C→T | *gltA → / ← yeiW* | citrate synthase/putative oxidoreductase |
| C→G | *gltA → / ← yeiW* | citrate synthase/putative oxidoreductase |
| A→C | *gltA → / ← yeiW* | citrate synthase/putative oxidoreductase |
| C→T | *gltA → / ← yeiW* | citrate synthase/putative oxidoreductase |
| +GGC | *PP_4387 ← / ← flgE* | unknown function/Flagellar hook protein |
| +GCC | *PP_4887 ← / ← PP_4888* | unknown function/chemotaxis transducer |
| +G | *rhlE‑II → / ← yceI* | RNA helicase/ stress response factor |
| G→C | *rhlE‑II → / ← yceI* | RNA helicase/ stress response factor |
| +CGGG | *PP_4986 ← / ← PP_4987* | Channel protein/Chemotaxis protein |
| T→C | *xpt → / ← PP_5266* | phosphoribosyltransferase/ acetyl‑CoA hydrolase |

#

# **Table S4. Occurrence of synonymous and non-synonymous convergent mutations in C16OH and C20OH variants.**

| Mutation | Locus Tag | Annotation | Occurrence in variants | | | | |
| --- | --- | --- | --- | --- | --- | --- | --- |
|  |  |  | C16OH-1 | C16OH-2 | C20OH-1 | C20OH-2 | C20OH-3 |
| C→T | *PP_0168* | S4900S (AGC→AGT) | Yes | Yes | Yes | Yes | Yes |
| G→A |  | Q4908Q (CAG→CAA) | Yes | Yes | Yes | Yes | Yes |
| T→C |  | G4909G (GGT→GGC) | Yes | Yes | Yes | Yes | Yes |
| C→T |  | V3353V (GTC→GTT) | Yes | Yes | Yes | Yes | Yes |
| C→A |  | Q3355N (CAG→AAC) | - | Yes | Yes | Yes | Yes |
| G→C |  | Q3355N (CAG→AAC) | - | Yes | Yes | Yes | Yes |
| (G)_5→4_ | *PP_1801* | coding (906/1143 nt) | Yes | Yes | Yes | - | - |
| G→T | *PP_1963* | A526S (GCC→TCC) | Yes | Yes | Yes | - | - |
| T→C | *PP_2331* | V468A (GTG→GCG) | Yes | Yes | Yes | - | - |
| A→G | *PP_2369* | F263L (TTC→CTC) | Yes | Yes | Yes | - | - |
| A→T | *PP_4896* | V466E (GTG→GAG) | Yes | Yes | Yes | - | - |
| G→A | *PP_5119 /*  *PP_5120* | intergenic (‑116/‑35) | Yes | Yes | Yes | - | - |

#

# **Table S5. Primers used in this work.**

| Name | Sequence 5’ to 3’ |
| --- | --- |
| pDONR_rev | *cgactctagaggatccccgggtac* |
| pDONR_for | *catcatgaaagcttggcactggcc* |
| ups_4760_for | tgcatgcctgcaggtcgacttgccggctgggtattctgacc |
| ups_4760_rev | cctcacttcacatgggtgggtctccgtatcagagc |
| ds_4760_for | cccacccatgtgaagtgagggcagggtagctg |
| ds_4760_rev | cggtacccggggatcctctaggcgaatgtgcgcgatctgct |
| ds_PP_5121/22_rev | aaatagcagacatacgaatgtcatgcgctgtgtgccaggcgg |
| ds_PP_5121/22_for | aagcttaggaggaaaaacatatgcaccgccgcgacctgctg |
| ups_PP_5121/22_for | ccggggatcctctagagtcgcaaaggcgcgcaagtactcgactt |
| ups_PP_5121/22_rev | gtgccaagctttcatgatgtcgaggaaatgaacgaataacgctccgacg |
| pS2313M_rev | atgtttttcctcctaagcttgcatgcctg |
| pS2313M_for | cattcgtatgtctgctatttcgcgtatagaactagtcttg |
| AlkL_for | aagcttaggaggaaaaacatATGAGTTTTTCTAATTATAAAGTAATCGCG |
| AlkL_rev | aaatagcagacatacgaatgTTACTAGAAAACATATGACGCACCAA |
